# Homogeneity Between Cervical and Vaginal Microbiomes and the Diagnostic Limitations of 16S Sequencing for STI Pathogens at Higher Ct Values

**DOI:** 10.3390/ijms26051983

**Published:** 2025-02-25

**Authors:** Claudio Neidhöfer, Mateja Condic, Nathalie Hahn, Lucia A. Otten, Damian J. Ralser, Nina Wetzig, Ralf Thiele, Achim Hoerauf, Marijo Parčina

**Affiliations:** 1 Institute of Experimental Haematology and Transfusion Medicine, University Hospital Bonn, 53127 Bonn, Germany; 2 Institute of Medical Microbiology, Immunology and Parasitology, University Hospital Bonn, 53127 Bonn, Germany; 3 Department of Gynecology and Gynecological Oncology, University Hospital Bonn, 53127 Bonn, Germany; 4 Institute for Functional Gene Analytics, Bonn-Rhein-Sieg University of Applied Sciences, 53757 Sankt Augustin, Germany

**Keywords:** cervico-vaginal microbiome, cervical microbiome, vaginal microbiome, sexually transmitted infections, STI diagnostics, *Ureaplasma parvum*, *Ureaplasma urealyticum*, *Mycoplasma hominis*, *Chlamydophila trachomatis*, 16S amplicon sequencing

## Abstract

Understanding the interactions between the cervico-vaginal microbiome, immune responses, and sexually transmitted infections (STIs) is crucial for developing targeted diagnostic and therapeutic strategies. Although microbiome analyses are not yet standard practice, integrating them into routine diagnostics could enhance personalized medicine and therapies. We investigated the extent to which partial 16S short-read amplicon microbiome analyses could inform on the presence of six commonly encountered STI-causing pathogens in a patient cohort referred for colposcopy, and whether relevant taxonomic or diagnostic discrepancies occur when using vaginal rather than cervical swabs. The study cohort included cervical and vaginal samples collected from women referred for colposcopy at the University Hospital Bonn between November 2021 and February 2022, due to an abnormal PAP smear or positive hrHPV results. 16S rRNA gene sequencing libraries were prepared targeting the V1–V2 and V4 regions of the 16S RNA gene and sequenced on the Illumina MiSeq. PCR diagnostics for common STI-causing pathogens were conducted using the Allplex STI Essential Assay Kit (Seegene, Seoul, Republic of Korea). Concerning the bacterial microbiome, no significant differences were found between vaginal and cervical samples in terms of prevalence of taxa present or diversity. A total of 95 patients and 171 samples tested positive for at least one among *Ureaplasma parvum*, *Ureaplasma urealyticum*, *Mycoplasma hominis, Mycoplasma genitalium*, *Chlamydophila trachomatis* or *Neisseria gonorrhoeae*. Sequencing the V1–V2 region enabled detection of one-third to half of the PCR-positive samples, with the detection likelihood increasing at lower cycle threshold (Ct) values. In contrast, sequencing the V4 region was less effective overall, yielding fewer species-level identifications and a higher proportion of undetermined taxa. We demonstrate that the vaginal microbiome closely mirrors the cervical microbiome, a relationship that has not been explored previously, but which broadens the possibilities for microbiome analysis and pathogen detection and establishes vaginal swabs as a reliable method for detecting the investigated pathogens, with sensitivities comparable with or superior to endocervical swabs. On the other hand, the sensitivity of partial 16S amplicon sequencing appears insufficient for effective STI diagnostics, as it fails to reliably identify or even detect pathogens at higher Ct values.

## 1. Introduction

The microbiome has garnered considerable attention and importance in recent years. However, despite its significance, comprehensive shotgun whole-genome analyses of the microbiome have yet to become standard practice. Instead, partial analyses utilizing various primer approaches are commonly employed [1]. Integrating microbiome analyses and similar methods into routine diagnostics could hold great potential for enhancing personalized medicine and enabling novel therapeutic approaches [2,3,4,5]. While most such applications are still in the research stage or early clinical trials, metagenomics sequencing for pathogen detection [6,7,8], and microbiome-based diabetes risk assessment are to some degree already in use [9,10], although the evidence base for the latter remains unresolved [11,12,13]. The vaginal microbiota is a dynamic ecosystem composed of more than 200 bacterial species but primarily dominated by *Lactobacillus*. It is influenced by factors such as genes, ethnic background, and environmental-behavioral factors and plays a crucial role in preventing infections and maintaining a low vaginal pH [14]. However, hormonal changes, such as those occurring during menopause, can alter the composition of the microbiota and increase the risk of conditions like bacterial vaginosis. The interplay between the vaginal microbiota, immune cells, and epithelial cells is essential for preventing infections and maintaining a healthy vaginal environment with sex hormones modulating pro-inflammatory cytokine expression, microbial composition, and thereby even antimicrobial peptide production [15]. By producing lactic acid, hydrogen peroxide, and bacteriocins, vaginal Lactobacillus species create an acidic and antimicrobial barrier that has been shown to inhibit *Neisseria gonorrhea*, *Trichomonas vaginalis*, and even HIV and HSV-1, among others [16,17,18,19,20]. Sexually transmitted infections (STIs) are not only influenced by the composition of the vaginal microbiota, immune responses, and epithelial homeostasis, but in turn themselves influence the very same biological processes, highlighting the importance of understanding these interactions for developing diagnostic and therapeutic strategies [15,21,22]. The most important bacterial STIs include *Chlamydia trachomatis*, a major pathogen responsible for non-gonococcal urethritis, cervicitis, and infertility; *Neisseria gonorrhoeae*, known for its mucosal infections and rising antibiotic resistance; and *Treponema pallidum*, the etiological agent of syphilis, a systemic disease with progressive stages and severe complications [23]. They are increasingly prevalent worldwide, driven by varied clinical presentations including asymptomatic cases, while rising antimicrobial resistance, particularly in *Neisseria gonorrhoeae* and *Mycoplasma genitalium*, poses significant challenges for diagnosis and treatment [24].

This study aimed to assess the extent to which short-read-based microbiome analysis can provide information comparable to targeted PCR diagnostics for six commonly encountered sexually transmitted bacterial pathogens, additionally focusing on distinctions between cervical and vaginal swabs. It builds upon previous research conducted in 2022/2023 on the cervical microbiome of women participating in the German national cervical cancer screening program, which explored the microbiome of HPV-positive women to identify potential correlations with dysplasia risk to define a role in preventive care [25]. No significant differences in bacterial microbiome diversity or taxa prevalence were found between vaginal and cervical samples, while sequencing the V1–V2 region detected one-third to half of PCR-positive cases.

## 2. Results

### 2.1. Participant and Sample Characteristics

The study cohort included 195 women from which cervical and vaginal microbiomes were available, totaling 390 samples. Age ranged from 20 to 78 averaging at 45.23, 139 women being pre- and 56 postmenopausal (see Table 1). After quality control, filtering, and chimera removal, we obtained an average of 18,239 reads per sample (range: 2334 to 68,219), totaling 7,113,653 reads across all samples.

### 2.2. General Comparison of Cervical and Vaginal Microbiomes (V1–V2)

Comparing the totality of vaginal and cervical samples, we found 16 out of the 160 most prevalent and evaluated taxa to display a higher prevalence in cervical samples as compared to vaginal ones. However, none were found to be significant after correcting for multiple testing using the Benjamini-Hochberg correction. No differences were found regarding sample richness or alpha-diversity (Shannon, Simpson, and Fisher).

### 2.3. PCR Results

A total of 95 patients tested positive for at least one of either *Ureaplasma parvum*, *Ureaplasma urealyticum*, *Mycoplasma hominis*, *Mycoplasma genitalium*, *Chlamydophila trachomatis*, or *Neisseria gonorrhoeae*. These were detected in twenty-eight cases only in vaginal swabs, in nine only in cervical swabs, and in sixty-seven cases in both, resulting in one hundred seventy-one samples that were positive for at least one pathogen (see Table 2). No sample tested positive for *Trichomonas vaginalis*, the only non-bacterial pathogen that would nonetheless potentially have been detected by the PCR test kit. Average Ct values were included to provide a general trend comparison.

*U. parvum* was detected in sixty patients both cervically and vaginally, in fifteen only vaginally, and in three only cervically, totaling one hundred thirty-eight samples positive for *U. parvum*. In cases where the bacterium was detected in both locations, the cycle threshold (Ct) value was significantly lower in the vaginal samples, which averaged 23.74 as compared to the cervical average of 27.94 (t (118) = −6.6, *p* =< 0.001). In cases in which it was only detected vaginally, the Ct value averaged 28.40, and when only detected cervically, the Ct values displayed an average of 28.79.

*U. urealyticum* was detected in eleven patients both cervically and vaginally, in nine only vaginally, and in one only cervically. In those cases where it was detected in both locations the Ct value was lower in vaginal samples, which averaged 25.96, as compared to the 27.71 average for cervical samples, although the difference was not statistically significant (t (20) = −1.16, *p* = 0.261). In cases in which it was only detected vaginally, the Ct value averaged 32.65.

*M. hominis* was detected in sixteen patients cervically and vaginally, in two only vaginally, and in one only cervically. In those cases where it was detected in both locations, the Ct value was significantly lower in vaginal samples, which averaged 20.39 as compared to the cervical average of 24.06 (t (25.93) = −3.36, *p* = 0.002).

*C. trachomatis* was detected in four patients both cervically and vaginally and in another four only vaginally. In those cases where it was detected only vaginally, the Ct value was higher (31.89 on average as compared to the cervical average of 26.92), and in those cases where it was detected in both locations each Ct value from the vaginal swabs was lower than the respective sample collected cervically. *M. genitalium* was detected in only one patient and only vaginally (Ct value 33.75). *N. gonorrhoeae* was detected in two patients and in both only vaginally (Ct values 26.53 and 33.37).

### 2.4. 16S Amplicon Sequencing

**Figure 1:**
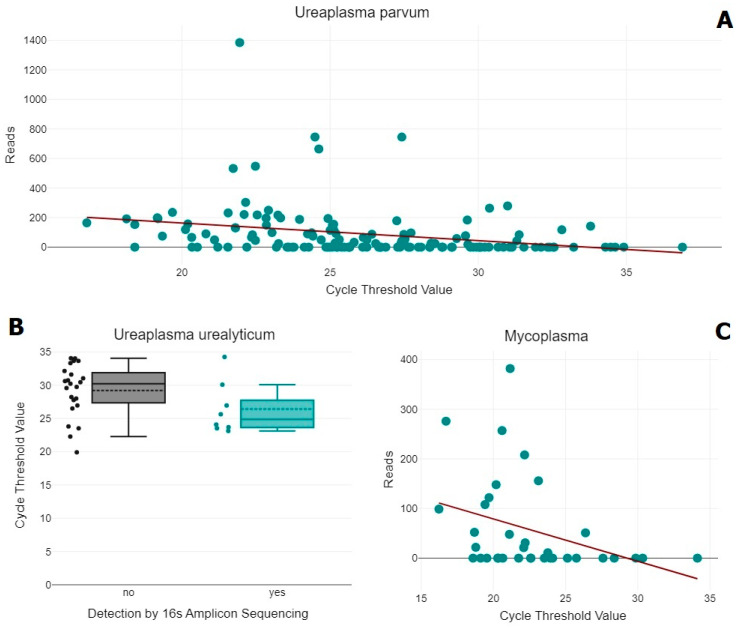
The scatter plots display the significant relationship between cycle threshold (Ct) values and read counts for *Ureaplasma parvum* (**A**) and *Mycoplasma* (**C**), with trend lines indicating the general correlation. The boxplot for *Ureaplasma urealyticum* (**B**) shows the Ct values categorized by detection status (yes/no) using 16S amplicon sequencing, highlighting the significantly differing distribution of Ct values in positive and negative detections.

In only 71 of 138 PCR-positive samples *U. parvum* was detected by 16S amplicon sequencing (AS) of the V1–V2 region (see Table 3). In one additional case, four reads assigned to the genus *Ureaplasma* were detected. The Ct value of the samples in which *U. parvum* was detected by 16S amplicon sequencing were significantly lower (t (136) = −4.92, *p* =< 0.001); in fact, there was a low, negative correlation between the number of detected reads and the Ct value (r (136) = −0.28, *p* = 0.001) (see Figure 1). The read number ranged from 19 to 1385 and averaged 174. In contrast, in two PCR-negative samples for *U. parvum*, 159 and 28 reads of it were detected. The number of detected reads did not differ significantly between vaginal and cervical samples (t (388) = 0.69, *p* = 0.492).

In only eight of thirty-two PCR-positive samples was *U. urealyticum* detected by 16S AS of the V1–V2 region. It was not detected in any PCR-negative samples. The Ct value did not correlate with the amount of detected reads; actual detection by 16S AS was however associated significantly with lower Ct values (t (29) = 1.73, *p* = 0.094). The read number ranged from 27 to 393, averaging 157.

Out of thirty-five PCR-positive samples for *M. hominis* detected by AS of the V1–V2 region, the *Mycoplasma* was detected at the genus level in sixteen cases, and additionally *M. hominis* was detected in five of these. In one PCR-negative sample, 23 reads of the genus *Mycoplasma* were detected. The read number of the remaining samples ranged from 33 to 253 for *M. hominis*, averaging 103, and 11 to 382 for the genus *Mycoplasma*, averaging 125. The read number of *Mycoplasma* (r (33) = −0.35, *p* = 0.038), but not *M. hominis* (r (33) = −0.17, *p* = 0.343), correlated moderately negatively with the Ct values. *M. genitalium* was detected in both samples of one patient who was only PCR-positive in the vaginal sample, with 104 reads vaginally and 40 reads in the cervical sample.

*C. trachomatis* was detected in only one instance through AS of the V1–V2 region, with 128 reads. Notably, this detection did not correspond to any of the 12 samples that had tested positive by PCR. *N. gonorrhoeae* was not detected in any sample at the species level, but it was detected in five samples at the genus level, none of which belonged to either of the two PCR-positive patients.

### 2.5. V1–V2 vs. V4

In total, 27 samples were additionally sequenced with a V4 primer pair to evaluate and compare key results to those of the V1–V2 primer pair; 15 were cervical and 12 were vaginal. Among the selected samples, twenty-one were PCR-positive for *U. parvum* but nineteen were undetected by AS of the V1–V2 region (19), three were PCR-positive for *U. urealyticum* but were undetected by AS of the V1–V2 region, two were PCR-positive for *M. hominis* but only one was found positive for the genus *Mycoplasma* by AS of the V1–V2 region, and only one sample was PCR-positive for *C. trachomatis*. Utilizing the V4 primer pair led to failure in detecting any of the aforementioned microorganisms at the species level. The genus *Ureaplasma* was detected in the two samples that were found positive for *U. parvum* by AS of the V1–V2 region, as well as in an additional eight samples that were PCR-positive for *U. parvum*, one of which was positive for *U. urealyticum* as well. *M. hominis* and *C. trachomatis* were detected at neither the genus nor family level.

**Figure 2:**
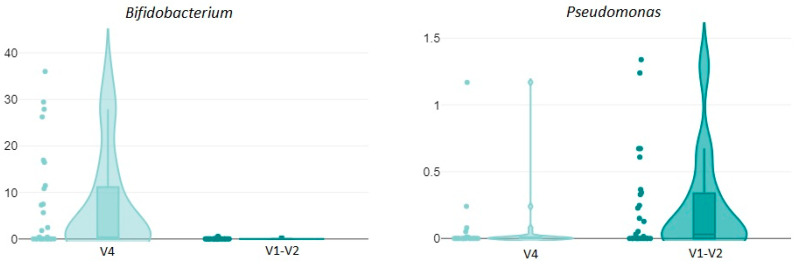
The violin plots display the significantly higher amount of *Bifidobacterium* detected when employing the primer pair targeting the V4 region as compared to the V1–V2 region, and the lower amount of detected *Pseudomonas*. On the y-axis is the respective organisms’ abundance in each sample.

The number of undetermined taxa at the species level averaged 52.03% for samples sequenced with the V4 primer pair as compared to 10.55% when sequencing the same samples with the V1–V2 primer pair (t (30.3) = 5.01, *p* =< 0.001). At the genus level, we looked at the five most prevalent taxa and found no difference for the amount of detected *Lactobacillus* (t (52) = 0.08, *p* = 0.935), *Prevotella* (t (31.75) = 1.7, *p* = 0.1), and *Streptococcus* (t (30.14) = −0.97, *p* = 0.338), but a much higher amount of *Bifidobacterium* with the V4 primer pair (t (26.01) = 3.52, *p* = 0.002) and a slightly higher amount of *Pseudomonas* with the V1–V2 primer pair (t (43.04) = −2.14, *p* = 0.038) (see Figure 2). Strikingly, even Shannon diversity differed significantly, being much higher when the V1–V2 region was targeted (t (37.22) = −8.83, *p* =< 0.001).

## 3. Discussion

Endocervical swabs have traditionally been the standard method for diagnosing STIs in women, but growing evidence now supports the use of vaginal swabs, including self-collected ones, as an equally reliable or even more effective option [26,27,28,29,30,31]. They demonstrate comparable, and in some cases even greater, sensitivity for most causative microorganisms, and are now recommended by the CDC as the most appropriate specimen type for detecting *C. trachomatis* and *N. gonorrhoeae* [32]. Our findings confirm that vaginal swabs reliably detect multiple STI-related pathogens with a sensitivity at least equal to that of endocervical samples. Notably, our study found that *U. parvum*, *U. urealyticum*, and *C. trachomatis* were more frequently detected in vaginal swabs and exhibited lower cycle threshold (Ct) values compared to cervical samples, likely indicating higher pathogen loads in the vagina than in the cervix. For *M. genitalium* and *N. gonorrhoeae*, sample size was too negligible to permit statements, yet both were detected exclusively in vaginal samples. Vaginal swabs offer a comfortable, non-invasive alternative that improves accessibility and patient acceptance, supporting the use of self-swabbing for private, convenient sample collection [29]. This approach expands access to STI testing, especially in resource-limited settings, and enhances compliance. It also opens up new possibilities for microbiome studies, given how our study highlights that the vaginal microbiome closely mirrors the cervical one, making vaginal swabs suitable for both pathogen detection and microbiome analysis of the cervico-vaginal environment.

The 16S amplicon sequencing (AS) method is commonly used to characterize the female genitourinary microbiome, as opposed to STI detection; as our study shows, its limitation are the inadequate species-level resolution when only short 16S rRNA gene segments are targeted and low sensitivity. Other targeted sequencing approaches, such as single-gene sequencing of pathogen-specific resistance markers, have shown more promising results [33]. In our study, when applying AS, only some pathogens were detected and only with limited effectiveness. Despite the fact that the DNA extraction step can undoubtedly compromise sensitivity for low-abundance taxa [34], our findings clearly demonstrate that the V4 region, in particular, is not a suitable primer for the mentioned diagnostic purpose. While it provided potentially better insights into the presence of *Bifidobacterium*, relevant for broader microbial landscape evaluations, and detected *Ureaplasma* at the genus level slightly better, the overall low species-level resolution limits its diagnostic utility. The V1–V2 region displayed better performance in comparison but still lacked the relevant sensitivity at low cycle threshold (Ct) values, and missed several pathogens. Although clinical symptoms might correlate with abundance [35], Ct values do not predict symptom likelihood or transmissibility for the investigated pathogens. Hence, the primary need would be to confirm their presence rather than quantifying them below a certain threshold. This indicates that as of now AS does not meet the diagnostic goal of reliably confirming pathogen presence and is not a viable option for the accurate detection of the studied microorganisms. Nucleic acid amplification tests (NAATs) remain the gold standard for STI detection due to their proven accuracy, yet even other novel approaches, which still require further validation, appear more applicable than AS of the V1–V2 or V4 region of the 16S rRNA gene for clinical STI diagnostics [33,36,37,38,39,40,41,42,43,44].

The reduced efficacy of AS for pathogen detection may result from primer bias, low microbial abundance, and sequencing depth limitations, leading to the under-detection of some variants. False negatives can occur due to insufficient template DNA or primer mismatches, while false positives may arise from index hopping, contamination, or misclassification [45,46]. PCR, though more sensitive, also carries risks of nonspecific amplification or reaction inefficiencies among others [47]. Comparing both methods to shotgun metagenomic sequencing would offer valuable insights into their relative accuracy and limitations.

The study largely focused on less significant microorganisms when compared to other more prominent STI-causing pathogens in terms of associated morbidity [35,48], which can be explained by the fact that our cohort did not consist of patients with STI-related symptoms but rather individuals undergoing HPV-related examinations. Consequently, our cohort does represent a sexually active population, and it did not prominently include individuals at an elevated STI risk. Microorganisms belonging to the genera *Ureaplasma* and *Mycoplasma* are, however, increasingly recognized for their clinical relevance in this context [25,49,50,51,52,53], making their identification indispensable in comprehensive STI screening, especially when other causes have been excluded. A final consideration addresses our reliance on the multiplex-PCR test as the reference method, which would certainly profit from closer inspection and assessment.

## 4. Materials and Methods

### 4.1. Study Cohort and Sample Collection

The study cohort included cervical samples from the previous study [25], collected from women who were referred for colposcopy to the certified Colposcopy Centre at the Department of Gynecology and Gynecological Oncology of the University Hospital Bonn from November 2021 until February 2022, indicated according to the guidelines of the new national cancer screening program (abnormal PAP smear finding and/or a positive result for hrHPV). The vaginal samples were collected from the vaginal canal by the same three experienced gynecologists in the same moment as the cervical ones, during the colposcopic examination, before the application of acetic acid, with a flocked swab (eNAT system, Copan Italia, Brescia, Italy).

### 4.2. Sample Preparation and Sequencing

Cervical and vaginal swabs were stored at 4 °C and subsequently processed within 2–9 days. Highly purified DNA was extracted from all samples using the column-based ZymoBIOMICS DNA Miniprep Kit (Zymo Research Europe GmbH, Freiburg, Germany). The isolation was performed strictly according to the manufacturer’s instructions. Mechanical lysis was performed with the Precellys^®^ Evolution homogenizer from Bertin Technologies SAS (Bretonneux, France). At the end of the extraction process, the DNA was eluted to a 100 uL volume and qualitatively and quantitatively evaluated using the NanoDrop OneC, Thermo Fisher Scientific Inc. (Waltham, MA, USA).

16S rRNA gene sequencing libraries were constructed from each sample using the Quick-16S NGS Library Prep Kit (Zymo Research Europe GmbH, Freiburg, Germany) with its included V1–V2 primer pairs, and from 28 selected samples with the Quick-16S Plus NGS Library Prep Kit (V4) (Zymo Research Europe GmbH, Freiburg, Germany). Each run included 94 samples, the positive control included in the kit, and a negative control. For quantitative PCR, quality control, and normalization purposes, the Bio-Rad CFX96 Real-Time PCR Detection System (Bio-Rad Laboratories, Inc., Hercules, CA, USA) was utilized.

After pooling, the DNA was quantified with the QuantiFluor dsDNA System on the Quantus Fluorometer (both: Promega GmbH, Walldorf, Germany) and diluted strictly according to the Illumina-protocol for MiSeq sample preparation. For the final V1–V2 library, a loading concentration of 10 pm was chosen and a 10% Illumina v3 PhiX spike-in control was added before running it on the Illumina MiSeq platform with a 500cycle v2 Illumina MiSeq Reagent Kit (all three: Illumina, San Diego, CA, USA).

Samples selected for resequencing with primers targeting the V4 region included those initially PCR-positive for *Ureaplasma parvum* (UP) but where the bacterium was undetected with the V1–V2 primer pair. For the final V4 library, a loading concentration of 4 pm was chosen and a 10% Illumina v3 PhiX spike-in control was added before running it on the Illumina MiSeq platform with a 300cycle v2 Illumina MiSeq Reagent Kit (all three: Illumina, San Diego, CA, USA).

### 4.3. Bioinformatic Analysis

The bioinformatic analysis included three main parts, starting with the preprocessing of raw paired-end reads. Following the preprocessing, the sequences were assigned to taxonomies. Finally, a statistical and graphical evaluation was performed on the resulting taxa. QIIME2 [54] was used for both preprocessing and classification of the data. With the plugin tool DADA2 [55], forward and reverse reads were trimmed from the 3′ end, while shorter than expected reads as well as low-quality reads were discarded. DADA2 was also used to perform error correction, merging of forward and reverse reads if there was an overlap of at least 12 base pairs, and chimera removal. The processed sequences were clustered into OTUs (operational taxonomic units) of 100% sequence identity, and assigned to taxa using a classifier trained on full-length sequences of SILVA [56]. The trained classifier was provided by QIIME2 using scikit-learn 0.24.1 and the plugin tool q2-feature-classifier [57,58].

### 4.4. PCR

For PCR STI diagnostics the Allplex STI Essential Assay Kit (Seegene, Seoul, Republic of Korea) was utilized, which enables simultaneous DNA testing for seven pathogens: *Mycoplasma hominis*, *Mycoplasma genitalium*, *Neisseria gonorrhoeae*, *Ureaplasma parvum*, *Ureaplasma urealyticum*, *Chlamydia trachomatis*, and the non-bacterial pathogen *Trichomonas vaginalis*. As an internal control had already been added to the extracted DNA in a previous study for microbiome analysis [25], the associated kit was not used here. Half the usual reagent and DNA volumes were used per PCR reaction, following an institute-validated approach to optimize resources and ensure reliable detection for research samples. For each PCR run, one field contained the mix with a positive control and another field was mixed with a negative control. The qPCR method was employed, with distinct fluorescence markers labeling each STI pathogen, allowing for the precise measurement of the bacterial load for each pathogen. The study analyzed 195 cervical and 195 vaginal samples, processed in batches of 94 samples each, alongside the mentioned controls.

### 4.5. Statistical Analysis

Statistical analysis was performed using Datatab version 1.12.1 for taxa frequency comparisons and correlation with clinical parameters. *p* values less than 0.05 were considered statistically significant.

### 4.6. Ethics Statement

The study was approved by the Ethics Committee of the Medical Faculty of the University of Bonn (vote: 128/21). All methods were carried out in accordance with relevant guidelines and regulations. Informed consent was obtained from all subjects.

## 5. Conclusions

This study demonstrates that the vaginal microbiome is largely homogeneous with the cervical microbiome, supporting the use of vaginal swabs as a reliable and non-invasive method for microbiome studies targeting that microenvironment or for detecting STI-related pathogens like *U. parvum*, *U. urealyticum*, *M. hominis*, and *C. trachomatis*. For pathogen-specific STI diagnostics, partial 16S rRNA sequencing lacked sufficient sensitivity, particularly at higher Ct values, limiting its applicability.

## Figures and Tables

**Table 1:** Patient Demographics.

Participants	195
Premenopausal	139
Postmenopausal	56
Age	45.23 (20–78)
Samples	390

**Table 2:** PCR results by sample types with average Ct values.

Pathogen	Total Positive Samples	Patients Detected Both Cervically and Vaginally	Patients Detected Vaginally Only	Patients Detected Cervically Only	Average Ct Value Across All Positive Vaginal Samples	Average Ct Value Across All Positive Cervical Samples
*U. parvum*	138	60	15	3	24.67	27.98
*U. urealyticum*	42	11	9	1	28.97	28.18
*M. hominis*	38	16	2	1	20.39	24.66
*C. trachomatis*	8	4	4	0	27.45	29.38
*N. gonorrhoeae*	2	0	2	0	29.95	-
*M. genitalium*	1	0	1	0	33.75	-

**Table 3:** Pathogen detection by 16S amplicon sequencing (AS) of the V1–V2 region.

Pathogen	PCR-Positive Samples Detected by 16S AS	PCR-Negative Samples Detected by 16S AS	Read Number Average (and Range)	Correlation with Ct Value
*U. parvum*	51.45% (71/138)	2 (28 and 159 reads)	174 (19–1385)	Low, negative (r (136) = −0.28, *p* = 0.001)
*U. urealyticum*	25.00% (8/32)	-	157 (27–393)	No correlation
*M. hominis*	14.29% (5/35)	-	103 (33–253)	No correlation
*C. trachomatis*	0/12	1	128	-
*N. gonorrhoeae*	0/2	-	-	-
*M. genitalium*	1/1	1	40 and 104	-

## Data Availability

The datasets generated during the current study are available from the corresponding author on reasonable request.
